# Multistep ctDNA Monitoring of Minimal Residual Disease in Colorectal Cancer Liver Metastases: From Tissue NGS to Highly Sensitive Digital PCR Platforms

**DOI:** 10.3390/diagnostics16050645

**Published:** 2026-02-24

**Authors:** Izabela Górzyńska, Agata Konieczka, Paweł Gaj, Michał Świerniak, Tomasz Stokłosa, Michał Grąt, Oskar Kornasiewicz

**Affiliations:** 1 Department of Tumor Biology and Genetics, Medical University of Warsaw, 02-091 Warsaw, Poland; izabela.gorzynska@wum.edu.pl (I.G.);; 2 Department of General, Transplant and Liver Surgery, Medical University of Warsaw, 02-097 Warsaw, Poland; 3 Warsaw Genomics, 02-106 Warsaw, Poland; 4 Laboratory of Genetics, University Clinical Centre, Medical University of Warsaw, 02-097 Warsaw, Poland

**Keywords:** liquid biopsy, ctDNA, digital PCR, colorectal cancer, liver metastases, minimal residual disease, disease recurrence, molecular monitoring

## Abstract

**Background/Objectives**: Colorectal cancer (CRC) liver metastases present a significant clinical challenge due to high recurrence risks post-resection. Traditional diagnostics often fail to detect early-stage minimal residual disease (MRD). This preliminary pilot study evaluated ctDNA dynamics in 10 patients with liver metastases using a personalized multistep approach. **Methods**: Following primary tumor Next-Generation Sequencing (NGS) to identify somatic mutations in *KRAS*, *NRAS*, *TP53*, *RET*, *APC*, and *WRN*, custom TaqMan assays were designed for longitudinal plasma analysis. Four methodologies were compared: HRM-PCR, PNA-enhanced qPCR, and two digital platforms (dPCR and ddPCR). **Results**: While HRM-PCR sensitivity was limited in plasma, digital platforms demonstrated 100% qualitative concordance. MRD-negative status (VAF 0.00%) was identified in 70% of cases (P01, P03, P06, P07, P08, P09, P10), while detectable ctDNA in patients P02, P04, and P05 strongly correlated with aggressive progression. Digital PCR enabled the ultra-low detection of Variant Allele Frequencies (VAFs), identifying high molecular burdens (e.g., P05, VAF 49%) correlating with rapid decline, and capturing early molecular residue in P04 (VAF 0.62%). **Conclusions**: Our preliminary findings confirm that personalized longitudinal VAF tracking via digital PCR provides superior prognostic value, serving as a robust tool for recurrence monitoring in personalized CRC therapy.

## 1. Introduction

Colorectal cancer (CRC) is currently the third most commonly diagnosed cancer both worldwide and in Poland [1]. Each year, approximately 20,000 new cases of colorectal cancer are diagnosed in Poland [2]. The majority of patients are diagnosed at the advanced stage IV of the disease. About 50% of CRC patients develop liver metastases (colorectal liver metastases—CRLM) during the natural course of the disease [3,4,5]. The presence of liver metastases in CRC is associated with poor prognosis, and the median survival for untreated disease ranges from six to twelve months [6]. Surgical resection of liver metastases is currently the only potentially curative treatment.

However, even after tumor resection, disease progression occurs in 50–60% of patients, even in those undergoing chemotherapy [4,5,7,8,9]. The latest data from the National Cancer Institute’s Surveillance, Epidemiology, and End Results (SEER) program indicate that the 5-year relative survival rate across all stages of CRC is 65.0% [8].

Postoperative monitoring includes radiological examinations (CT, MRI) [10,11] and the measurement of tumor markers such as alpha-fetoprotein (AFP) [12], carcinoembryonic antigen (CEA) [13], and Ca19-9 antigen [14]. The most widely used risk assessment system is the Clinical Risk Score (CRS), which considers the number and size of metastases, lymph node status of the primary tumor, preoperative CEA levels, and the disease-free interval between removal of the primary tumor and the appearance of metastases (<12 months) [13]. Moreover, the diagnostic accuracy and sensitivity of pathological and imaging methods for detecting disease progression or identifying molecular minimal residual disease (MRD) are limited, while the specificity and diagnostic utility of serum markers (e.g., CEA) remain low [15]. Imaging techniques such as CT, MRI, and PET—used in standard monitoring protocols—increase the likelihood of detecting recurrence but are not sensitive enough to detect small lesions, particularly in patients after liver resection or treatment with modern chemotherapy, such as targeted therapies [16,17].

Most patients do not have measurable disease on imaging [18] until a clear recurrence becomes apparent. Therefore, there is an urgent need to develop new methods for long-term, non-invasive tumor monitoring and for predicting recurrence significantly earlier than with standard imaging techniques. Additionally, such methods are needed to evaluate the effectiveness of surgical interventions and chemotherapy, especially in stage IV CRC. Liquid biopsy is a modern, minimally invasive diagnostic method based on the analysis of body fluid samples—such as blood, urine, or other biological fluids—to detect tumor markers that may indicate the presence of cancer. It currently serves as an alternative to traditional biopsy, which requires the collection of tumor tissue samples. This technique is becoming one of the main areas of research in precision oncology. Over the past decade, it has gained significant popularity due to its ability to provide information about the tumor in a way that is easier and less burdensome for the patient [19,20].

It is crucial to note that ctDNA is a specific subset of the total cell-free DNA (cfDNA) pool. While cfDNA is present in all individuals and originates primarily from the apoptosis of normal hematopoietic cells, ctDNA is found only in cancer patients and carries tumor-specific somatic mutations. During the laboratory process, total cfDNA is isolated from the plasma, and the ctDNA fraction—which may constitute less than 1% of the total isolated DNA—is subsequently identified and quantified using highly sensitive molecular techniques [21,22]. Previous studies have shown that ctDNA levels in a patient’s blood are strongly correlated with tumor burden and cancer stage [23,24,25]. Additionally, patients with stage I colorectal cancer exhibited significantly lower ctDNA levels compared to those with stage IV disease, further confirming the relationship between ctDNA quantity and tumor size [26,27,28]. Based on these findings, it can be assumed that ctDNA analysis may serve as a molecular link between the primary tumor and metastases, reflecting disease progression. It may also provide a reference point for tumor staging and significantly improve existing methods for recurrence monitoring [29].

The exact mechanism of ctDNA release into the bloodstream is not yet fully understood. The most likely processes include apoptosis and necrosis of tumor cells, as well as active DNA release by living cells. Tumor cells may undergo apoptosis (programmed cell death) in response to stress, chemotherapy, radiotherapy, or as a natural consequence of uncontrolled cell growth. During apoptosis, cells disintegrate and release DNA fragments into the extracellular space, from which they enter the bloodstream. Research has shown that the length of ctDNA is typically around 166 base pairs, corresponding to the length of DNA wrapped around a nucleosome with a short linker—suggesting that apoptosis is the main mechanism of ctDNA release [30,31]. Another significant mechanism is necrosis, an uncontrolled form of cell death that occurs due to mechanical or toxic damage—such as the effects of anticancer drugs, ischemia (tumor-associated blood flow obstruction), or other factors. During necrosis, the cell breaks down and releases its contents, including DNA, into the bloodstream [32].

## 2. Study Design

This study was designed as a prospective assessment of the impact of somatic mutations on colorectal cancer progression in patients following liver metastasis resection. Based on the tumor-specific variants identified through NGS, selected genetic alterations were subsequently monitored in post-operative blood samples using four complementary molecular techniques: high-resolution melting PCR (HRM-PCR), quantitative PCR (qPCR), digital droplet PCR (ddPCR), and digital PCR (dPCR).

## 3. Materials and Methods

### 3.1. Sample Collection

Between August and December 2022, 10 patients diagnosed with metastatic colorectal cancer underwent tumor resection surgery at the Department of General, Transplant and Liver Surgery, Medical University of Warsaw. The sample collection protocol was designed to provide a comprehensive molecular profile for each patient, starting with peripheral blood drawn into standard EDTA tubes one day prior to surgery for the isolation of germline genomic DNA (gDNA). During the surgical procedure, representative tumor tissue samples were harvested for somatic mutation profiling. Subsequent longitudinal monitoring of circulating tumor DNA (ctDNA) was conducted through follow-up blood collections at 4 weeks and 3 months post-resection, utilizing Roche Cell-Free DNA Collection Tubes to stabilize nucleated cells and prevent the release of genomic DNA into the cell-free DNA (cfDNA) fraction.

### 3.2. Next-Generation Sequencing (NGS)

To establish a patient-specific genetic map, gDNA was isolated from the pre-operative EDTA blood samples using the Maxwell^®^ RSC Blood DNA Kit (Promega, Madison, WI, USA), while tumor DNA was extracted from 50 mg of resected tissue using the Invisorb Spin Tissue Mini Kit (Invitek Molecular, Berlin, Germany). Genomic libraries were prepared with the KAPA HyperPlus Kit (Roche Sequencing Solutions, Pleasanton, CA, USA). Whole coding sequences of genes associated with tumorigenesis (e.g., *KRAS*, *NRAS*, *TP53*, *RET*, *APC*, and *WRN*) were sequenced on Illumina NextSeq500 or NovaSeq4000 platforms, (Illumina, San Diego, CA, USA) achieving an average sequencing depth of 95.4× for gDNA and 320.5× for tissue. Raw sequencing reads were aligned to the human reference genome (GRCh38) using the Burrows–Wheeler Aligner (BWA). Somatic variant calling was performed using the GATK (Genome Analysis Toolkit, Broad Institute, Cambridge, MA, USA) and Mutect2 algorithm. Variants were filtered based on a minimum coverage depth of 100× and an allele frequency (AF) > 1%, with clinical significance verified against ClinVar and COSMIC databases. Finally, a comparative bioinformatic analysis of both materials was performed to identify tumor-specific variants and construct a tumor genome map.

### 3.3. Plasma Processing and cfDNA Isolation

Plasma separation from the stabilized Roche tubes was performed using a two-step centrifugation protocol: an initial spin at 1600× *g* for 10 min at room temperature, followed by high-speed centrifugation of the plasma fraction at 16,000× *g* for 10 min to eliminate residual cellular debris. The resulting cell-free plasma was aliquoted and stored at −80 °C until cfDNA extraction [33,34,35].

The cfDNA was isolated using the cobas^®^ cfDNA Sample Preparation Kit (Roche Sequencing Solutions, Pleasanton, CA, USA). To minimize contamination from high-molecular-weight genomic DNA and enrich for the mononucleosomal cfDNA fraction (approximately 167 bp), an additional double-sided size selection was performed using AMPure XP beads (Beckman Coulter, Brea, CA, USA). In the first step, a 0.6× bead-to-sample ratio was used to bind and remove large DNA fragments. The supernatant, containing the short DNA fraction, was subsequently transferred to a new tube where the bead ratio was adjusted to 1.2× to capture the target cfDNA. After two washes with 80% ethanol, the enriched cfDNA was eluted in nuclease-free water, quantified, and stored at −20 °C until downstream molecular analysis [36,37].

### 3.4. High-Resolution Melting PCR (HRM-PCR)

Tumor-specific genetic variants identified by NGS were used to design primers for HRM-PCR analysis, according to previously published protocols [38]. Primer sequences are provided in Table 1. HRM-PCR reactions were performed using the LightCycler^®^ 480 High-Resolution Melting Master (Roche Sequencing Solutions, Pleasanton, CA, USA). Each reaction was prepared in a final volume of 15 µL, containing 2× High-Resolution Melting Master Mix, 1.2 µL of a 10 µM primer mix, 1 µL of genomic DNA (approximately 40 ng) or 5 µL of cfDNA.

Amplification and melting analyses were carried out on a LightCycler^®^ 480 system (Roche Sequencing Solutions, Pleasanton, CA, USA). PCR conditions included initial denaturation at 95 °C for 3 min, followed by 50 cycles of denaturation at 95 °C for 10 s and annealing at 60 °C for 60 s. Melting curve analysis was conducted with incubation at 95 °C for 60 s and 40 °C for 60 s, followed by a gradual temperature increase from 75 °C to 90 °C at a rate of 0.2 °C every 10 s. Data were analyzed using LightCycler^®^ 480 Software version 1.5 with the Gene Scanning module.

### 3.5. Quantitative PCR (qPCR)

Quantitative PCR was performed using the cobas^®^ *KRAS* Mutation Test (Roche Molecular Diagnostics, CA, USA), applied exclusively to samples from patients in whom pathogenic *KRAS* mutations were previously identified by NGS analysis of tumor tissue. This targeted assay is designed for the sensitive detection of clinically relevant mutations in codons 12, 13, and 61 of the *KRAS* gene [39,40]. All reactions were conducted on the LightCycler^®^ 480 system (Roche) in accordance with the manufacturer’s protocol [39]. The assay enabled both qualitative and semi-quantitative assessment of the *KRAS* mutation status in post-operative plasma-derived ctDNA

### 3.6. Digital PCR Analysis: ddPCR and dPCR Platforms

Absolute quantification of ctDNA was achieved using the QIAcuity Digital PCR (dPCR) system (Qiagen, Hilden, Germany) as the primary quantification platform, complemented by the QX200 Droplet Digital PCR (ddPCR) system (Bio-Rad Laboratories, Hercules, CA, USA) for methodological robustness and cross-platform validation [41,42]. Custom-made TaqMan Genotyping Assays (Thermo Fisher Scientific, Waltham, MA, USA) were specifically designed for each patient, utilizing a dual-probe system with FAM for mutant and VIC for wild-type alleles to enable high-precision tracking of tumor-specific variants [43].

#### 3.6.1. Analytical Validation and Limit of Detection (LOD)

To determine the analytical sensitivity of the personalized assays, a validation study was performed using a KRAS G12A synthetic control. A serial dilution series (1:1 ratio) was prepared in a background of wild-type genomic DNA to simulate clinical conditions. The thresholds for defining positive partitions were established for each assay using a combination of No Template Controls (NTC) and Wild-Type (WT) DNA samples to define the limit of blank (LoB) and prevent false-positive calls. The Limit of Detection (LOD) was defined as the lowest mutant allele concentration that could be consistently identified across replicates. Linearity was assessed by calculating the Pearson correlation coefficient (R2) between theoretical and measured VAF values. This validation was primarily conducted on the QIAcuity system, with selected points verified on the QX200 platform to assess cross-technology concordance [44].

#### 3.6.2. Digital PCR (dPCR)—Primary Platform

The QIAcuity platform was utilized for lead quantification due to its nanostructured Nanoplate 26k architecture, which provides approximately 26,000 partitions per well. This high degree of compartmentalization significantly enhances the signal-to-noise ratio, enabling the detection of ultra-low-frequency mutations [45]. The 40 µL reaction mixture was prepared using the QIAcuity LNA PCR Probe Kit and combined with personalized TaqMan Genotyping Assays. The nanoplates were processed in an integrated QIAcuity instrument, which automates partitioning, thermocycling, and multi-channel imaging. The thermal profile included: initial activation at 95 °C for 2 min, followed by 40 cycles of denaturation at 95 °C for 15 s and annealing/extension at 60 °C for 30 s. Data were processed using the QIAcuity Software Suite (version 3.1, Qiagen, Hilden, Germany) where thresholds were meticulously established to ensure clear cluster separation between FAM and VIC channels. Manual adjustments in the QIAcuity Software Suite (version 3.1, Qiagen, Hilden, Germany) were applied when automatic classification was suboptimal to ensure precise differentiation between positive and negative partitions.

#### 3.6.3. Droplet Digital PCR (ddPCR)—Methodological Validation

To provide independent orthogonal validation, samples were cross-analyzed using the QX Manager Software (version 2.2 Standard Edition, Bio-Rad Laboratories, Hercules, CA, USA) [46]. Each 20 µL reaction mixture contained 10 µL of ddPCR Supermix for Probes (No dUTP) and 1 µL of the same personalized TaqMan Genotyping Assay used in the dPCR workflow. The mixture was partitioned into approximately 20,000 monodisperse droplets. Thermal cycling was performed in a C1000 Touch™ Thermal Cycler: initial denaturation at 95 °C for 10 min, 40 cycles of denaturation at 94 °C for 30 s and annealing/extension at 51 °C for 1 min, with a final stabilization at 98 °C for 10 min. A ramp rate of 2 °C/s was maintained to ensure droplet integrity. Results were processed via QuantaSoft™ Analysis Pro. Thresholds were set following the same validation principles as the dPCR platform, utilizing NTC and WT controls to ensure cross-platform consistency. The Variant Allele Frequency (VAF%) was calculated based on Poisson distribution statis-tics in accordance with the Digital MIQE guidelines [47], using the ratio of mutant-positive partitions to the total number of valid partitions (VAF = MutMut + WT × 100%).

This dual-platform approach, leveraging both fixed-partition (QIAcuity) and oil-emulsion (QX200) technologies [48], ensured the high reproducibility of molecular residual disease (MRD) detection and validated the ctDNA dynamics observed throughout this study [45,46].

## 4. Results

### 4.1. NGS Mutational Profiling and ctDNA Characterization

This study began with a comprehensive mutational profiling of 10 patients with colorectal cancer liver metastases (CRLMs). By comparing germline DNA from preoperative blood with somatic DNA from resected tumor tissue via Next-Generation Sequencing (NGS), we identified a range of tumor-specific variants. These mutations, primarily affecting the *KRAS*, *NRAS*, *TP53*, *APC*, and *RET* genes, provided the necessary molecular targets for personalized longitudinal monitoring. The identified pathogenic and potentially pathogenic variants are summarized in Table 2.

**Figure 1:**
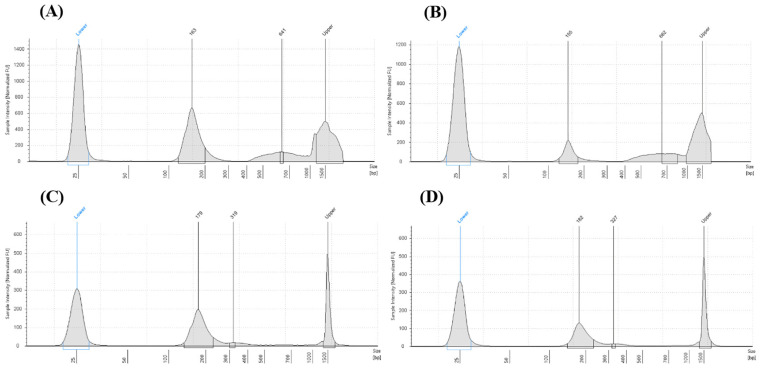
cfDNA isolation quality visualized on TapeStation electropherogram. Comparison of cfDNA quality of two separate patients before the purification procedure (**A**,**B**) and after purification (**C**,**D**). In both cases, the size selection protocol successfully removed high-molecular-weight genomic DNA (gDNA) contamination, enriching the mononucleosomal fraction. Abbreviations: bp, base pairs; cfDNA, cell-free DNA; gDNA, genomic DNA.

Following surgery, cfDNA was isolated from plasma samples at two time points. Quantitative assessment showed that for the majority of the cohort, cfDNA concentrations increased between the 4-week and 3-month assessments (Table 3). The quality of the isolated DNA was verified using the TapeStation 4200 analyzer (Agilent Technologies, Santa Clara, CA, USA), which confirmed the presence of characteristic ctDNA fragments at approximately 134 bp and 144 bp (Figure 1) [49]. Larger DNA fragments identified during analysis were categorized as genomic DNA (gDNA) contaminants, likely resulting from minor erythrocyte hemolysis or leukocyte lysis during sample handling [50]. To maintain high analytical sensitivity, the double-sided size selection protocol was successful in enriching the mononucleosomal cfDNA fraction for downstream digital PCR analysis.

### 4.2. Comparative Performance of Traditional PCR Platforms

**Figure 2:**
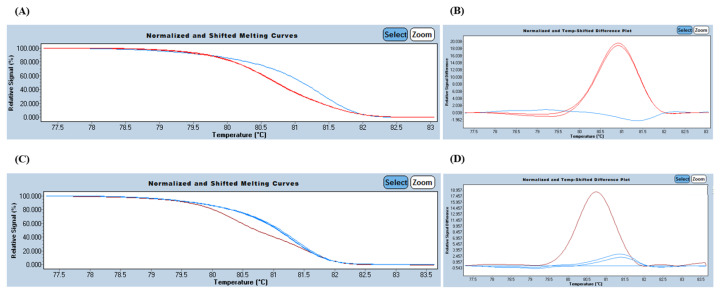
Results for the variants NM_004985.5:c.35G>T and NM_004985.5:c.35G>A in the *KRAS* gene. On the left, the melting curve is shown, and on the right, the temperature difference plot is displayed. (**A**,**B**) Graphs showing the presence of the NM_004985.5:c.35G>T variant in the patient P05 ctDNA. (**C**,**D**) Graphs showing the absence of the NM_004985.5:c.35G>A variant in the patient P06 ctDNA. Red curve—heterozygote, blue curve—homozygote. Abbreviations: ctDNA, circulating tumor DNA; HRM-PCR, high-resolution melting polymerase chain reaction.

Preliminary screening of the 19 plasma samples using HRM-PCR revealed significant limitations in the liquid biopsy setting. Although the custom-designed HRM primers successfully confirmed mutations in 97% of the tumor tissue samples, the method demonstrated a high rate of false-negatives in plasma. A representative positive result was observed only for patient P05, where a high molecular burden allowed for a distinct shift in the melting profile (Figure 2). However, for patients with lower tumor burdens, such as P02, the HRM-PCR analysis failed to distinguish the mutant signal from the wild-type background, while patient P06 consistently showed a wild-type melting profile (Figure 2).

**Figure 3:**
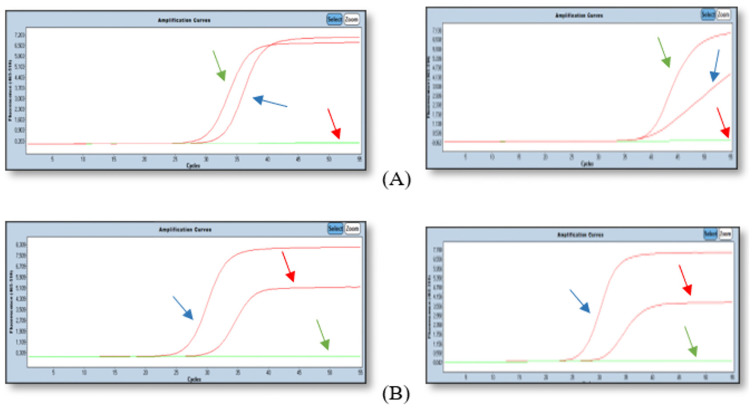
Qualitative ctDNA assessment using the *KRAS* Mutation Test v2 (Roche Sequencing Solutions, Pleasanton, CA, USA). The graphs on the left show the positive control results: (**A**) NM_004985.5:c.35G>A and (**B**) NM_004985.5:c.35G>T. The graphs on the right show the test results for the patient’s P06 (**A**) and P05 (**B**) ctDNA. Arrow colors indicate: green arrows for wild-type amplification curves, blue arrows for mutant-type amplification curves, and red arrows for internal control or baseline thresholds. Abbreviations: ctDNA, circulating tumor DNA; qPCR, quantitative polymerase chain reaction.

To further validate the initial screening, the commercial KRAS Mutation Test v2 (Roche Sequencing Solutions, Pleasanton, CA, USA) was applied to the subset of four patients (P02, P05, P06, and P07) in whom NGS had previously identified *KRAS* hotspot mutations. The results varied across the patients and time points. For patient P02, the test yielded a negative result at the 4-week mark but successfully detected a *KRAS* mutation in the 3-month post-operative sample (Figure 3), reflecting an increase in ctDNA levels over time. In contrast, the personalized dPCR approach described in the following section identified a VAF of 0.74% for P02 at the same 4-week mark, highlighting a significant lead-time advantage of approximately two months over the commercial qPCR standard. In the case of patient P05, the single available plasma sample showed a strong positive signal due to the exceptionally high initial molecular burden (Figure 3). In contrast, for patients P06 and P07, the assay failed to detect any *KRAS* mutations in either the 4-week or 3-month samples, remaining consistently negative.

Ultimately, while the commercial test outperformed HRM-PCR, it proved insufficient for the broader cohort. It was inherently limited to the four patients with *KRAS* mutations, leaving the six patients with other variants (e.g., *APC*, *TP53*, *RET*) without a viable monitoring tool. A key limitation of this real-time PCR-based assay is its qualitative nature; it provides a binary “Detected/Not Detected” result for specific codons rather than identifying the exact nucleotide substitution or calculating the Variant Allele Frequency (VAF%). Consequently, while the test can indicate a mutation within a specific codon, it lacks the resolution to distinguish between different variants in that locus or to quantify the molecular burden.

### 4.3. Digital PCR Comparative Performance of dPCR and ddPCR Platforms

#### 4.3.1. Analytical Validation: Linearity and Sensitivity Thresholds

To ensure the highest level of clinical reliability, a rigorous analytical validation of the primary QIAcuity dPCR platform was conducted. This process was based on a serial dilution series of a synthetic KRAS G12A standard within a background of wild-type (WT) genomic DNA. Linear regression analysis demonstrated exceptional correlation (*R*^2^ = 0.969) between the theoretical and measured Variant Allele Frequency (VAF) values across a broad dynamic range.

A critical component of this validation was the determination of the practical Limit of Detection (LOD). The QIAcuity system successfully identified the mutant variant down to a 0.12% VAF threshold, corresponding to the detection of 4 mutant copies in the presence of 7054 wild-type copies. At a lower concentration of 0.06% VAF, the signal was no longer reproducible, allowing for the definition of a stringent analytical cutoff for clinical samples.

**Figure 4:**
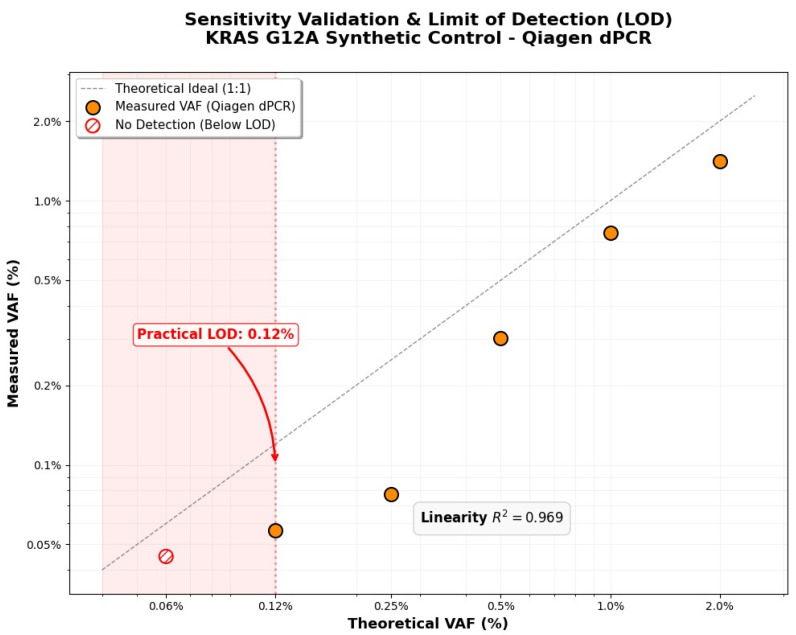
Sensitivity validation and Limit of Detection (LOD) analysis using a KRAS G12A synthetic control on the QIAcuity dPCR platform. The plot illustrates the correlation between theoretical and measured VAF values (*R*^2^ = 0.969), with the practical LOD established at 0.12%. Points below 0.12% (red) indicate the threshold where consistent detection was no longer achievable. Abbreviations: dPCR, digital polymerase chain reaction; LOD, limit of detection; VAF, variant allele frequency.

This high degree of sensitivity is a direct result of the Nanoplate 26k architecture; by partitioning the sample into thousands of independent reaction chambers, the system minimizes the inhibitory effect of the wild-type DNA background and significantly enhances the signal-to-noise ratio (SNR). As shown in Figure 4, the measured values followed the theoretical ideal (1:1 ratio) until reaching the established LOD threshold.

#### 4.3.2. Comparative Assessment of dPCR and ddPCR in Clinical Samples

At the 4-week postoperative assessment, ctDNA was detected in three patients: P02, P04, and P05. Patient P05 demonstrated an identical, exceptionally high molecular burden of 49.05% VAF on both platforms. This high tumor burden correlated with a poor clinical prognosis, as the patient passed away before the 3-month follow-up could be performed.

**Figure 5:**
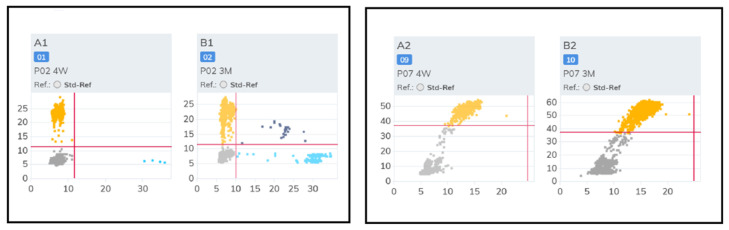
Representative dPCR scatter plots from the QIAcuity Software Suite illustrating personalized ctDNA assessment. The presence of molecular residual disease (MRD) is characterized by distinct clusters: light blue partitions (containing only mutant alleles) and dark blue partitions (double-positive partitions containing both mutant and wild-type alleles). The dominant yellow cluster represents the wild-type (WT) background. The partitions are restricted exclusively to the wild-type DNA population (yellow) and empty partitions (grey). The horizontal and vertical red lines indicate the fluorescence thresholds used to rigorously distinguish true positive signals from baseline noise, ensuring the high analytical precision of the VAF% calculation. Abbreviations: dPCR, digital polymerase chain reaction; MRD, molecular residual disease; VAF, variant allele frequency; WT, wild-type.

**Figure 6:**
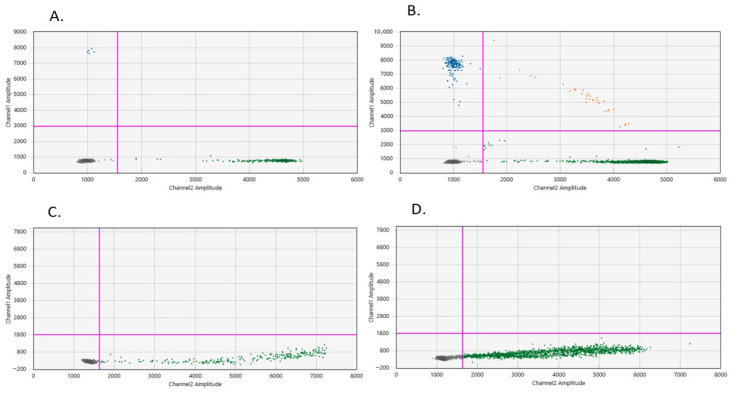
Representative 2D droplet amplitude plots from the Bio-Rad QX200 ddPCR (Bio-Rad Laboratories, Hercules, CA, USA). (**A**,**B**) Initial detection of molecular residual disease (MRD), showing early clusters of mutant-positive (blue) and double-positive (orange) droplets. Clinical progression is reflected by a significant increase in the density of mutant-containing clusters (blue and orange). (**C**) A molecularly negative status, with droplets localized exclusively in the wild-type (green) and negative (grey) zones. (**D**) Continued molecular response, confirming the absence of detectable ctDNA. The horizontal and vertical magenta lines indicate the fluorescence thresholds used for partition classification. Abbreviations: ddPCR, droplet digital polymerase chain reaction; MRD, molecular residual disease; ctDNA, circulating tumor DNA.

For patient P02, dPCR detected a VAF of 0.74%, while ddPCR yielded a value of 1.83%. The 3-month assessment revealed critical molecular progression in a subset of the cohort. Patient P02 showed a significant increase in VAF to 5.21% (dPCR) and 5.13% (ddPCR). This progression is visually captured in both Figure 5 (dPCR) and Figure 6 (ddPCR). A notable comparison was performed for Patient P04 (*RET* variant). At the 4-week time point, both platforms showed high quantitative agreement, with a VAF of 0.62% via dPCR and 0.59% via ddPCR. However, a divergence was observed at the 3-month time point, where dPCR captured a rise in molecular burden to 1.96%, while the ddPCR result remained relatively stable at 0.60%. This divergence, further illustrated in the analytical panels Figure 5(A1,B1) and Figure 6A,B, may be attributed to technical variability near the limit of detection and differences in partition volume between the fixed-partition and droplet-based systems. Crucially, despite these quantitative differences, both platforms consistently provided the same qualitative clinical result, identifying Patient P04 as MRD-positive at both time points.

Patients P01, P03, P06, P07, P08, P09, and P10 remained consistently ctDNA-negative (0.00% VAF) on both platforms throughout the study period. This molecularly negative status is illustrated in Figure 5(A2,B2) and Figure 6C,D, where partitions and droplets are localized exclusively in the wild-type and negative zones, confirming sustained molecular remission. Overall, the higher VAF values and clearer cluster separation obtained via dPCR in low-concentration samples suggest that the QIAcuity platform may offer enhanced efficiency in capturing rare mutant alleles.

#### 4.3.3. Clinical Correlation and Lead-Time Advantage over Standard Methods

The most significant finding of the comparative analysis is the drastic difference in sensitivity between the personalized digital approach and routine laboratory diagnostics. Conventional HRM-PCR proved insufficient for ctDNA monitoring, identifying only the single case with an exceptionally high concentration (P05). The results for patient P02 are particularly clinically relevant. The commercial qPCR assay (Roche Sequencing Solutions, Pleasanton, CA, USA), used as a diagnostic standard, failed to detect the presence of the mutation 4 weeks post-surgery (“Not Detected”). During the same time point, dPCR analysis allowed for the quantification of MRD at 0.74% VAF. A positive signal in the commercial test did not appear until two months later, by which time the tumor burden had increased to over 5%.

This two-month “lead-time” offered by dPCR technology represents a critical therapeutic window that, in clinical practice, could allow for earlier treatment modification before radiological symptoms appear.

Furthermore, the personalized dPCR approach enabled the monitoring of patients with rarer mutations (e.g., the *RET* variant in patient P04) that fall outside the “hotspot” panels of commercial assays. This ensured 100% molecular coverage of the study cohort, making dPCR a significantly more versatile tool for personalized medicine. Overall, these results suggest that longitudinal ctDNA monitoring provides a high-resolution window into the patient’s molecular response and serves as a powerful tool for early risk stratification. A comprehensive summary of the molecular findings across all tested platforms, including tissue mutational status, dPCR/ddPCR results, and the final clinical outcomes for each patient, is presented in Table 4. This integrated dataset highlights the high concordance between personalized assays and the clinical course of the disease. This two-month difference in detection between dPCR and commercial qPCR for Patient P02 represents a significant gain in sensitivity. In our cohort, the longitudinal dynamics of ctDNA VAF showed high qualitative consistency with the clinical course of the disease. For patients who experienced disease progression (e.g., P01, P02, and P04), a persistent or rising molecular signal was observed. While the current study did not include a synchronized side-by-side comparison with every radiological time point, the observed molecular trends aligned with the aggressive clinical outcomes, suggesting that dPCR-based monitoring could serve as a valuable complementary tool to standard clinical assessments.

### 4.4. Longitudinal Surveillance and Survival Analysis

**Figure 7:**
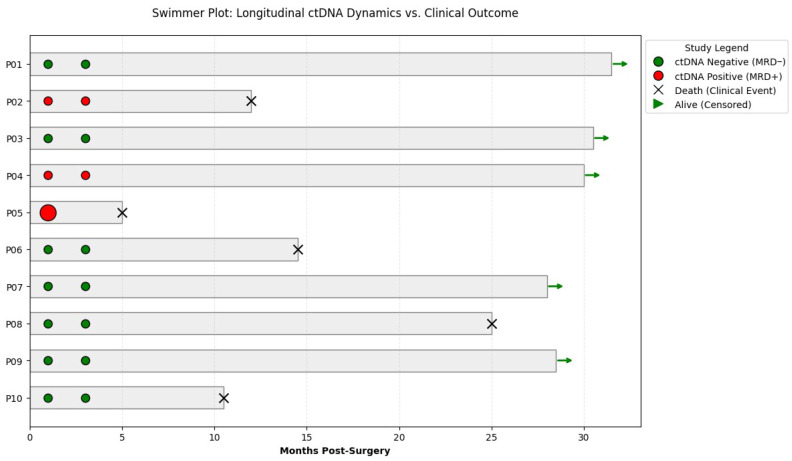
The swimmer plot illustrates the individual clinical courses of patients P01–P10 relative to their longitudinal ctDNA status. The horizontal bars represent overall survival time in months, starting from the surgical procedure. Markers placed at the 4-week and 3-month time points indicate the results of liquid biopsy monitoring, where green circles represent ctDNA-negative status (MRD-) and red circles signify ctDNA-positive status (MRD+), with the circle size reflecting the Variant Allele Frequency (VAF%). Clinical outcomes are denoted at the end of each bar, using a black ’X’ to mark mortality and a green arrow to indicate patients who were alive and censored at the last follow-up. Abbreviations: MRD, molecular residual disease; VAF, variant allele frequency.

To evaluate the clinical utility of ctDNA monitoring as a predictive marker for disease recurrence and survival, we integrated the longitudinal mutational data with patient clinical outcomes in a Swimmer Plot (Figure 7). This analysis aimed to determine whether the detection and dynamics of ctDNA post-resection could serve as a surrogate indicator for rapid clinical progression or molecular relapse. The clinical trajectories of the study cohort revealed a strong correlation between Molecular Residual Disease (MRD) status and overall survival. Patients exhibiting persistent ctDNA positivity (P02, P05) experienced significantly shorter survival times, with clinical events (death) occurring shortly after the detection of high or rising Variant Allele Frequency (VAF%) levels. Specifically, patient P05 demonstrated a high molecular burden (49.05% VAF) shortly after surgery, which correlated with rapid disease progression and death within 5 months.

Patient P02 also showed a rising molecular trend (from 0.74% to 5.21% VAF), with death occurring at approximately 12 months post-surgery. These cases highlight the high positive predictive value of personalized dPCR assays for identifying patients at extreme risk of recurrence. Conversely, a consistently negative ctDNA status across all monitored time points (P01, P03, P06, P07, P08, P09, and P10) was associated with varying clinical outcomes but generally longer survival in the majority of the cohort. Patients P01, P03, P07, and P09 demonstrated prolonged survival exceeding 30 months post-surgery, underscoring the high negative predictive value of the assay for long-term remission. Although patients P08 and P10 remained ctDNA negative, clinical events were recorded at 25 and 10 months respectively, suggesting that in these specific cases, recurrence may have been driven by factors outside the tracked molecular variants or below the current limit of detection. Notably, patient P06 remained ctDNA negative throughout the surveillance period, which distinguishes this case from the high-risk positive group, despite the eventual clinical event at 14 months. This highlights that clinical trajectories can vary between individuals. In contrast, patients in the “Other Mutations” group (e.g., P01, P03, P09) showed significantly prolonged survival, with most surviving beyond 30 months. event at 14 months. Interestingly, patient P04 remains alive at the 30-month mark despite persistent ctDNA positivity. However, the rising molecular trend captured by the primary dPCR platform (from 0.62% to 1.96%) indicates a state of molecular progression that war-rants continued clinical surveillance. Overall, these results suggest that longitudinal ctDNA monitoring provides a high-resolution window into the patient’s molecular response and serves as a powerful tool for early risk stratification.

#### Correlation Between Mutational Status and Overall Survival

**Figure 8:**
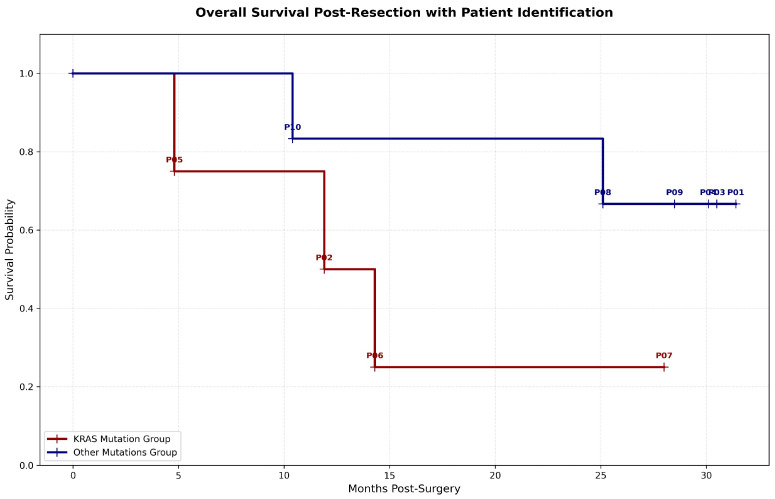
The Kaplan–Meier plot illustrates the probability of survival over time for patients divided into two cohorts: the KRAS mutation group (red line) and the “Other Mutations” group (blue line). Cross symbols on the curves indicate censored data points (patients alive at the last follow-up), while each vertical drop represents a clinical event (death). Individual patient IDs are annotated at the time of their respective event or censoring point. A clear separation in survival probability is observed, with the KRAS mutation group exhibiting a steeper decline in survival. Abbreviations: KRAS, Kirsten rat sarcoma virus gene; ID, identifier.

To further evaluate the clinical impact of the identified molecular profiles, a survival analysis was performed comparing patients based on their primary tumor mutational status. The inclusion of an Overall Survival (OS) curve with patient-specific identification (Figure 8) allows for a clear visualization of the correlation between genetic drivers, ctDNA dynamics, and clinical prognosis.

Our findings underscore that the presence of a KRAS mutation acts as a significant negative prognostic factor in this cohort. As shown in the survival plot, patients harboring KRAS variants (represented by the red line) exhibited markedly shorter overall survival compared to those in the “Other Mutations” group (blue line). Specifically, patients P05, P02, and P06, all of whom carried pathogenic KRAS mutations, reached terminal clinical endpoints significantly earlier—within 5 to 15 months post-resection.

Interestingly, while the KRAS group generally showed a higher correlation with detectable molecular residual disease (MRD) and rapid clinical deterioration (e.g., P05 with 49.05% VAF), patient P06 remained ctDNA-negative throughout the longitudinal monitoring despite the presence of a tissue KRAS mutation. This suggests that while tissue mutational status is a strong prognostic indicator of poor outcomes, ctDNA shedding may

## 5. Discussion

Colorectal cancer (CRC) remains one of the most prevalent malignancies worldwide, and its metastatic spread to the liver continues to represent a major therapeutic challenge. Despite significant advances in surgical techniques and systemic chemotherapy, a substantial proportion of patients experience disease recurrence, often several years after curative-intent resection. This clinical reality poses an ongoing challenge for oncologists, particularly in the postoperative monitoring of patients following liver metastasis resection and in the detection of molecular residual disease (MRD) [1,2,3,4,5,6,7,8,9].

Traditional surveillance strategies, including imaging modalities such as CT, MRI, and PET, as well as serum biomarkers like carcinoembryonic antigen (CEA), play an essential role in clinical practice but suffer from limited sensitivity in detecting occult micrometastatic disease and in predicting recurrence at an early stage. Consequently, there is an urgent clinical need for novel, more sensitive, and more specific tools capable of detecting disease recurrence earlier and enabling real-time assessment of surgical and systemic treatment efficacy [10,11,12,13,14,15,16,17].

In this context, the emergence of liquid biopsy approaches based on the analysis of circulating tumor DNA (ctDNA) has opened new avenues for non-invasive cancer monitoring. In the present study, we evaluated the clinical and technical utility of ctDNA analysis in patients with metastatic CRC to the liver (CRLM), comparing multiple molecular platforms, including next-generation sequencing (NGS), HRM-PCR, qPCR, and digital PCR technologies. Our findings indicate that ctDNA monitoring can outperform traditional imaging techniques, providing molecular evidence of disease recurrence weeks or even months before radiological lesions become detectable. For patients demonstrating molecular persistence or recurrence (e.g., P01, P02, and P04), the detection of a rising VAF trend aligned with their clinical deterioration. Although a precise lead-time analysis compared to imaging was not performed due to the pilot nature of the study and the retrospective character of clinical data collection, our results are qualitatively consistent with the general trends observed in larger trials, such as the GALAXY study [51]. These data reinforce the concept that molecular monitoring provides a highly sensitive window into the patient’s status, potentially supporting clinical decision-making alongside traditional surveillance methods. To the best of our knowledge, this is one of the few studies to offer such a comprehensive head-to-head comparison of multiple diagnostic modalities. While many studies focus on a single technology, our multi-platform approach provides a unique perspective on the analytical hierarchy required for effective MRD monitoring. Recent studies, such as those by Henriksen et al. (2023) and Jiang et al. (2023) [52,53] have further reinforced the prognostic value of ctDNA in the CRLM (Colorectal Liver Metastases) setting. However, our work extends these findings by directly evaluating the practical transitions between traditional PCR and advanced digital platforms.

Comprehensive NGS analysis of primary tumor tissue constituted the cornerstone of our diagnostic strategy. Our approach aimed to target pathogenic variants with high clinical significance documented in public databases (ClinVar, COSMIC). However, it is important to note that NGS results do not always yield well-characterized “driver” mutations for every patient. In such cases, the selection was based on the most robust somatic variants available in the individual’s profile. While this tumor-informed strategy is recognized as the gold standard for MRD detection [54,55], it is not without limitations. By focusing on primary tissue variants, we may not capture new mutations emerging through clonal evolution during metastasis or treatment. This could lead to false-negative results if the tracked variant is no longer representative of the total tumor burden, a challenge that future studies using broader panels or “agnostic” approaches may help to address. The mutations identified in our cohort included alterations in genes related to chromosomal instability and oncogenic signaling pathways, most notably *KRAS* and *APC*, which are well-established drivers of colorectal carcinogenesis. In particular, KRAS mutations—especially in the context of liver metastases—are recognized as strong prognostic markers and are associated with poorer treatment outcomes. Recent clinical data from the CIRCULATE-Japan project confirm that KRAS-mutated CRLM patients exhibit a distinct molecular recurrence pattern and may derive different levels of benefit from adjuvant therapies compared to wild-type cohorts [56].

In the second phase of the study, ctDNA detection was performed using HRM-PCR assays. While HRM-PCR successfully confirmed mutations in 97% of tumor tissue samples, ctDNA detection in plasma was achieved in only a single patient. This pronounced discrepancy underscores the extremely low abundance of ctDNA following surgical resection and highlights the inherent limitations of conventional PCR-based approaches. Recent methodological assessments confirm that while HRM-PCR is cost-effective for tissue screening, its analytical sensitivity (typically 1–5%) is insufficient for the post-resection setting, where variant allele frequencies (VAF) often drop below 0.1% [57]. From a technical standpoint, ctDNA analysis in the MRD setting is inherently challenging due to the extremely low concentration of circulating cell-free DNA (cfDNA) recovered from plasma. In many cases, the total genomic input is near the theoretical limits of detection, requiring meticulous laboratory protocols to avoid sample loss. As highlighted by many experts, the “stochastic effect” at ultra-low concentrations makes traditional PCR prone to false-negative results [58]. This scarcity of template DNA necessitates the use of ultra-sensitive technologies like dPCR, as conventional methods fail to distinguish the minute tumor signal from the overwhelming background of wild-type DNA derived from healthy cells [59,60].

A key technical finding of this study was the superior performance of digital PCR platforms. The high quantitative agreement observed in cases like Patient P04 at the first follow-up (0.62% vs. 0.59%) validates the robustness of both systems. Discrepancy observed at the 3-month point for this specific patient (1.96% vs. 0.60%) likely reflects technical variability near the limit of detection and differences in partition volume between fixed-partition and droplet-based systems. Crucially, despite these quantitative differences, both platforms consistently provided the same qualitative clinical result, identifying the patient as MRD-positive. Notably, the QIAcuity dPCR system (Qiagen, Hilden, Germany) emerged as particularly advantageous, offering a more streamlined work-flow and a more cost-effective profile compared to the droplet-based Bio-Rad system.

Recent independent evaluations of the QIAcuity nanoplate-based system confirm that its automated partition generation reduces hands-on time and minimizes the risk of sample cross-contamination, which is critical for clinical MRD workflows [61,62]. Furthermore, as summarized in Table 4, the integration of digital PCR platforms with longitudinal monitoring allowed for the precise tracking of molecular trends, particularly in patients like P02 and P04, where rising VAF% levels served as a direct indicator of disease persistence.

However, our study also highlights the biological complexity of ctDNA shedding. A significant observation was made in the case of Patient P06. Despite harboring a pathogenic KRAS mutation in the primary tumor tissue, Patient P06 remained consistently ctDNA-negative across all postoperative time points. These biological challenges of “low-shedding” tumors are consistent with recent observations by Abidoye et al. (2025) [63]. Several mechanisms may underlie this phenotype: liver metastases can exhibit different growth patterns (e.g., desmoplastic vs. pushing) that influence DNA release. Furthermore, factors such as poor tumor vascularization, a high stromal-to-epithelial ratio, or lower metabolic activity in metastatic lesions may limit the release of ctDNA into the systemic circulation, potentially affecting the sensitivity of liquid biopsy even when clinical imaging remains positive. This suggests a “non-shedding” phenotype, where the molecular burden remains below the limit of detection even in the presence of aggressive disease, a phenomenon reported in up to 15–20% of metastatic CRC cases [64].

This phenomenon was also observed in Patient P10, underscoring that while a positive ctDNA result (MRD+) is highly predictive of recurrence, a negative result (MRD-) does not definitively exclude the presence of residual disease. This reinforces the findings of the GALAXY study [55], which demonstrated that although ctDNA is a powerful prognostic marker, clinical decisions should ideally integrate tissue-based genomic profiling (Figure 8), longitudinal ctDNA analysis, and conventional imaging to capture the full clinical picture.

### Clinical Implications and Proof of Concept

This study should be clinically regarded as a “ preliminary pilot Proof of Concept”. The relatively small cohort size reflects the significant logistical challenges associated with longitudinal clinical research in the CRLM setting. Obtaining high-quality clinical material and maintaining consistent contact with patients after hospital discharge to secure longitudinal plasma samples is a major hurdle in postoperative monitoring research.

Despite these limitations, our results support the clinical utility of ctDNA-based monitoring. While the small sample size precludes definitive conclusions regarding survival probabilities, our results support the qualitative clinical utility of ctDNA-based monitoring. The high negative predictive value observed in patients like P01 and P07 (Table 4) indicates that consistent ctDNA negativity is a strong indicator of prolonged survival. Conversely, the case of P06 underscores the necessity of a multi-modal approach, integrating tissue-informed genomics, liquid biopsy, and conventional imaging (Figure 8) to minimize the risk of false-negative evaluations.

## 6. Conclusions

In this preliminary pilot study, we demonstrated that a personalized, tumor-informed ctDNA monitoring strategy using digital PCR (dPCR and ddPCR) provides a high-resolution window into molecular residual disease (MRD) in patients with colorectal liver metastases. Our multi-platform comparison highlights that while traditional PCR methods (HRM-PCR and commercial qPCR) are effective for tissue screening or detecting high molecular burdens, they lack the sensitivity required for effective postoperative surveillance.

The results indicate a strong qualitative alignment between rising ctDNA Variant Allele Frequency (VAF%) levels and clinical disease progression. Although the small cohort size and the retrospective nature of clinical data collection preclude definitive survival analysis or the calculation of a precise radiological lead-time, the observed molecular trends suggest that longitudinal ctDNA tracking can serve as a powerful complementary tool to standard-of-care imaging.

Furthermore, we identified biological challenges such as the “low-shedding” phenotype and the potential for clonal evolution, which must be considered when interpreting negative ctDNA results. Overall, our findings support the clinical utility of personalized digital PCR assays for early risk stratification and suggest that this approach could significantly enhance personalized therapeutic strategies in the CRLM setting. Future larger-scale prospective trials are warranted to fully quantify the clinical impact and standardized implementation of this technology.

## Figures and Tables

**Table 1:** Primers Used in High-Resolution Melting—Gene Scanning and Genotyping Methods.

Gene	dbSNP ID	Forward Primer (5′→3′)	Reverse Primer (5′→3′)	Size (bp)
*ALK*	rs1442742511	GCTGGGAGGCGCGAATTG	GCTGTTCAGTTGGTGGATTCGCC	122
*APC*	rs137854574	TGAGGAATTTGTCTTGGCG	TAACTTCTAAAGCACATTCCATC	96
	rs397515734	GCGAAGAATAGCCAGAATTC	AGGCAATTTACTAACCTCTGC	100
	rs1064793022	GGAGTACCTTAACATGATGT	TCGAGGTGCAGAGTGTGTGCTAC	148
	rs62619935	CCTGAGCTTTTAAGTGGTAG CCAT	CTGCTTCTGTTGCTTGGGAC TGTA	173
	rs1114167577	CTGCAGTTCAGAGGGTCCAG	AATGGCTCATCGAGGCTCAG	124
*KRAS*	rs121913530	TTGGATCATATTCGTCCACAA	AGGCCTGCTGAAAATGACTG	116
*NRAS*	rs121913254	TGGCAAATACACAGAGGAAGC	TGGTGAAACCTGTTTGTTGG	110
*PIK3CA*	rs121913287	GTTACTCAAGAAGCAGAAAG	CGGTTGCCTACTGGTTCAATTAC	107
*RET*	rs576743947	GTGAGGCCCCTCCTGCCCAGC	TCGCTCTGCTTCTCTAGGC	114
*TP53*	rs11540652	GTGGCAAGTGGCTCCTGAC	GCTCTGACTGTACCACCATCCAC	125
	rs11575997	TGAGTGTTAGACTGGAAACT	CAACAACACCAGCTCCTCTC	120
	rs28934578	GCCCTGTCGTCTCTCCAG	CGCGCCATGGCCATCTAC	120
	rs28934576	CGGAGATTCTCTTCCTCTGTGCGCC	AGAAAGGACAAGGGTGGTTGGGAG	195
	rs757274881	CAGCTGCTCACCATCGCTATCTG	TCTTCCTACAGTACTCCCCTGCC	206

**Table 2:** Pathogenic and potentially pathogenic variants identified in next-generation sequencing. These variants were selected based on previously chosen criteria described in the main text.

Patient No.	*KRAS* Mutation	Other Identified Variants
P01	–	rs121913254||rs62619935||rs757274881
P02	G12C	rs1114167577||rs28934576
P03	–	rs200855118
P04	–	rs576743947
P05	G12V	rs397515734||rs11575997
P06	G12D	rs121913287
P07	G12D	rs202246266
P08	–	rs137854574||rs587782329
P09	–	rs28934578||rs1442742511
P10	–	rs11540652||rs1064793022||rs62619935

**Table 3:** Summary of the obtained ctDNA quantity from the patients’ plasma. * Sample unavailable due to rapid clinical progression.

Patient No.	First Draft	Second Draft
P01	40 ng	28 ng
P02	40 ng	98 ng
P03	77 ng	42 ng
P04	28 ng	42 ng
P05	70 ng	N/A *
P06	42 ng	105 ng
P07	28 ng	77 ng
P08	42 ng	49 ng
P09	35 ng	52.5 ng
P10	56 ng	112 ng

**Table 4:** Comparative analysis of ctDNA detection across diagnostic platforms. Positive results (VAF > 0 or detected) are highlighted in bold.

Patient HRM-PCR Commercial qPCR dPCR (Primary) ddPCR (Validation) Outcome									
ID	4W	3M	4W	3M	4W (%)	3M (%)	4W (%)	3M (%)	
P01	.	.	N/A	N/A	.	.	.	.	Alive
P02	.	.	.	**Det.**	**0.74%**	**5.21%**	**1.83%**	**5.13%**	Death
P03	.	.	N/A	N/A	.	.	.	.	Alive
P04	.	.	N/A	N/A	**0.62%**	**1.96%**	**0.59%**	**0.60%**	Alive
P05	**Det.**	N/A	**Det.**	N/A	**49.05%**	N/A	**49.05%**	N/A	Death
P06	.	.	.	.	.	.	.	.	Death
P07	.	.	.	.	.	.	.	.	Alive
P08	.	.	N/A	N/A	.	.	.	.	Death
P09	.	.	N/A	N/A	.	.	.	.	Alive
P10	.	.	N/A	N/A	.	.	.	.	Death

**Det.** = Detected; **.** = Not Detected; **N/A** = Not Applicable; **VAF** = Variant Allele Frequency.

## Data Availability

The data presented in this study are available on request from the corresponding author due to privacy and ethical restrictions.

## Abbreviations

The following abbreviations are used in this manuscript:

bp	Base pairs
BWA	Burrows–Wheeler Aligner
CEA	Carcinoembryonic antigen
cfDNA	Cell-free DNA
CRC	Colorectal cancer
CRLM	Colorectal cancer liver metastases
ctDNA	Circulating tumor DNA
ddPCR	Droplet Digital Polymerase Chain Reaction
dPCR	Digital Polymerase Chain Reaction
EDTA	Ethylenediaminetetraacetic acid
GATK	Genome Analysis Toolkit
gDNA	Genomic DNA
HRM-PCR	High-Resolution Melting Polymerase Chain Reaction
KRAS	Kirsten rat sarcoma virus oncogene
LOD	Limit of Detection
MRD	Molecular Residual Disease
NGS	Next-Generation Sequencing
OS	Overall Survival
qPCR	Quantitative Polymerase Chain Reaction
VAF	Variant Allele Frequency
WT	Wild-Type
